# Dissecting combining ability effect in a rice NCII-III population provides insights into heterosis in *indica-japonica* cross

**DOI:** 10.1186/s12284-017-0179-9

**Published:** 2017-08-29

**Authors:** Hao Zhou, Duo Xia, Jing Zeng, Gonghao Jiang, Yuqing He

**Affiliations:** National Key Laboratory of Crop Genetic Improvement and National Centre of Plant Gene Research, Huazhong Agricultural University, Wuhan, China

**Keywords:** *Oryza sativa* L﻿., Combining ability, Mating design, *indica-jaonica* cross, Linkage analysis, Heterosis

## Abstract

**Background:**

Combining ability is a measure for selecting elite parents that make the highest contributions to hybrid performance. However, the genetic bases of combining ability and how they contributed to heterosis is seldomly known.

**Results:**

We constructed a both NCII and NCIII population derived from an *indica-japonica* cross to study the relationship among parental performance, combining ability and hybrid performance of 11 agronomic traits. Among them, specific combining ability is more important to grain yield than parental performance and general combining ability. We performed linkage analyses to phenotypic values and combining ability of all 11 traits in Doubled haploid lines and its two backcross populations and identified 108 QTLs in total. Among these QTLs, four known loci, *Sd1*, *Ghd7*, *Ghd8* and *DEP1* contribute a lot to GCA effects of agronomic traits except grain yield and seed setting rate. Three QTLs, *Ghd8*, *S5* and *qS12*, contribute a lot to SCA effects of grain yield and present overdominace.

**Conclusions:**

Our study provides insights into the genetic bases of combining ability and heterosis and will promote the improvements of *indica-japonica* hybrid breeding.

**Electronic supplementary material:**

The online version of this article (10.1186/s12284-017-0179-9) contains supplementary material, which is available to authorized users.

## Background

The phenomenon heterosis has been applied to rice breeding for improving grain yield since the early 1970s in China (Cheng et al. 2007; Darwin 1876; Li et al. 2007). To breed an ideal cross with the highest grain yield and great stress resistance is the ultimate goal of hybrid rice breeders. As parents with excellent agronomic traits do not always pass those traits on to their progenies, breeders often test the potential of a selfing line by cross it to several other lines. The potential for creating high heterosis progenies of an inbred line is called combining ability, a concept proposed by Sprague and Tatum and has been widely used in cross breeding for elite parent selection (Comstock and Robinson 1948; Griffing 1956; Sprague and Tatum 1942). Parent lines with high general combining ability (GCA) in grain yield and resistance to pests and diseases are more likely to form hybrids with satisfactory performance. On the other hand, some parent lines of general GCA also is able to form excellent hybrids and this is caused by another special effect called special combining ability (SCA). The both effects of GCA and SCA are able to create hybrids with high heterosis but the genetic bases of these effects are largely unknown.

The first attempt to unveil the genetic basis of combining ability was done by Griffing (1956). He proposed the methods of using diallel-cross to dissect the genetic variance into additive variance and non-additive variance, and estimated the GCA and SCA effects. His study provide theory basis for estimating genetic variance and combining ability effect in various mating design, including complete or incomplete diallel (Griffing 1956), North Carolina design (Comstock et al. 1949), and top crossing (Hill et al. 1998). Later study conducted transcriptome analysis and molecular markers to reveal the relationship between combining ability and genetic diversity (Ajmone et al. 1998; Bernardo 1996; Frisch et al. 2010; Stupar et al. 2008). Those studies revealed that the genetic distance between two parents are positively correlated with combining ability and hybrid performance.

Recently, with the development of molecular marker, linkage analysis has been used to dissect the GCA effects into quantitative trait locus (QTL) (Lv et al. 2012; Qi 2013; Qu 2012). Liu et al. (2015) utilized a NCII design, performed linkage analyses to GCA, and confirmed that *Ghd7* and *OsPRR37* (Koo et al. 2013; Xue et al. 2008) are major genes for GCA of heading date, plant height and spikelet per panicle in rice. Though a few QTLs contributed to combining ability has been indentified, the genetic bases of combining ability are still not clear and how they contributed to hybrid performance was totally unknown. In this study, we developed a both NCII and NCIII population (see Methods) derived from an *indica-japonica* cross, performed linkage analysis to both GCA and SCA effects, and explored how QTLs of combining ability contributed to hybrid performance of hybrid rice.

## Results

### The design of a both NCII and NCIII population

**Fig. 1:**
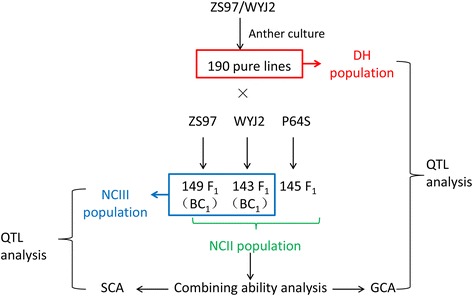
The experimental design and analysis procedure used in this study

The F_1_ generation of an *indica* line ZS97B (ZS97) and a *japonica* line Wuyugeng2 (WYG) has heterosis in PH, HD, KGW and SP, but has hybrid weakness in SS (Table 1). This hybrid sterility phenomenon leads to the lower YD of F_1_ than both parents. To dissect the genetic basis of the heterosis and hybrid weakness of ZS97 × WYG, we constructed the both NCII and NCIII population using the doubled haploid lines (DHs) of ZS97 × WYG as male parents and taking the two parents and P64S as female parents (Fig. 1). By crossing the 190 DHs to the three tester lines, we finally get 149 F_1_ for ZS97 × DHs, 143 F_1_ for WYG × DHs and 145 F_1_ for P64S × DHs.

**Table 1:** The performances of an *indica-japonica* cross in 11 agronomic traits

	GF	PH	HD	YD	TP	KGW	SP	GP	SS	PL	SDEN
ZS97	90.7	79.3	69.5	23.1	12.7	21.7	109.6	83.7	76.4	19.5	5.6
WYG	99.6	84.2	99.0	44.7	16.8	24.9	132.0	107.1	81.2	15.9	8.3
F_1_	99.7	121.1	109.0	22.6	14.2	27.0	147.4	58.8	39.9	19.9	7.4

*GF* grian-filling degree, *PH* plant height, *HD* heading date, *YD* yield, *TP* number of tillers per panicle, *KGW* grain weight per 1000 grains, *SP* spikelet per panicle, *GP* number of grains per panicle, *SS* seed-setting rate, *PL* panicle langth, *SDEN* grain density per panicle

### Performance of the populations.

**Fig. 2:**
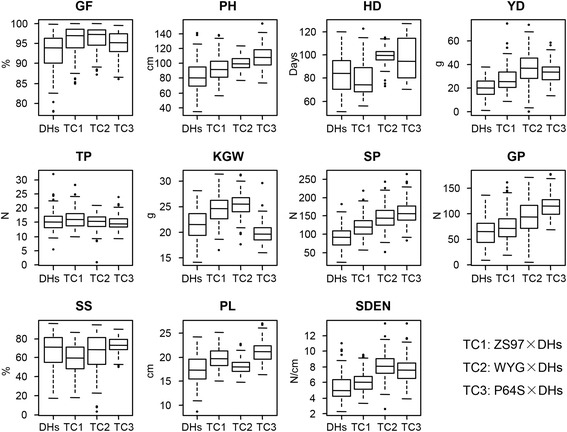
The phenotypic distributions of 11 agronomic traits in the DH population and their TC progenies. GF, grian-filling degree; PH, plant height; HD, heading date; YD, yield; TP, number of tillers per panicle; KGW, grain weight per 1000 grains; SP, spikelet per panicle; GP, number of grains per panicle; SS, seed-setting rate; PL, panicle langth; SDEN, grain density per panicle

The phenotypic distributions of 11 agronomic traits measured in the DHs and their TC progenies are shown in Fig. 2. All these traits varied widely in the DHs and three TC populations and most of these traits showed normal distributions expect GF and SS.

The average levels of most traits, except HD, KGW and SS, in the TC populations were higher than that in the DHs. The average HD in DHs was higher than that of ZS97 × DHs, while higher than that of the other two TC populations. The average SS in ZS97 × DHs and WYG × DHs were both lower than that in DHs. These differences indicate that the three TC populations might have a high level of special combining ability and heterosis.

### Relationship between traits values in DHs and TC populations

Table 1 shows the correlations among the phenotypic values and GCA effects of DHs, the phenotypic values and SCA effects of TC populations for the 11 agronomic traits. The phenotypes of DHs are significantly and positively correlated with GCA effects of DHs in most traits expect YD and SS. This indicated the genetic bases of GCA and trait value for YD and SS may be quite different. Both GCA and SCA effects are positively correlated with phenotypic values of all traits in all three TC populations, indicates that both GCA and SCA contribute a lot to hybrid performance.

### Variance analysis of combining ability

We determined the effects of GCA (DHs or Testers) and SCA (Testers × DHs) by two-factor variance analysis and found these effects were significant in all traits. This also indicated that both kinds of gene effects were important for the inheritance of agronomic traits. We further calculated the additive variance (V_A_), non-additive variance (V_Na_) and narrow sense heritability (*h* ^*2*^_*N*_) for each trait. These traits with *h* ^*2*^_*N*_ larger than 0.5 indicates they are mainly controlled by additive effects, as PH, HD and KGW; conversely, those with *h* ^*2*^_*N*_ below 0.5 are mainly controlled by non-additive effects, as GF, YD and SS. This also indicates over-dominance contribute a lot to the heterosis of GF, YD and SS.

### QTL mapping for combining ability

**Fig. 3:**
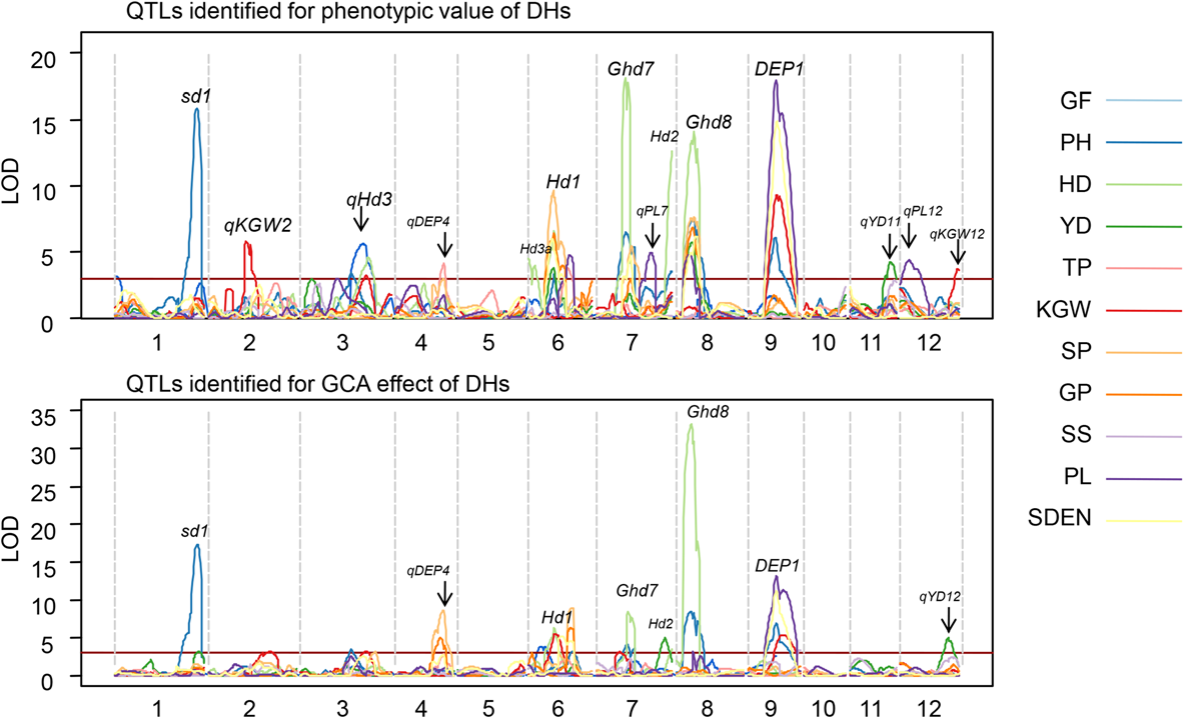
The linkage mapping of phenotypic values and GCA effect for 11 agronomic traits in DHs. For a description of agronomic traits see materials and methods

**Fig. 4:**
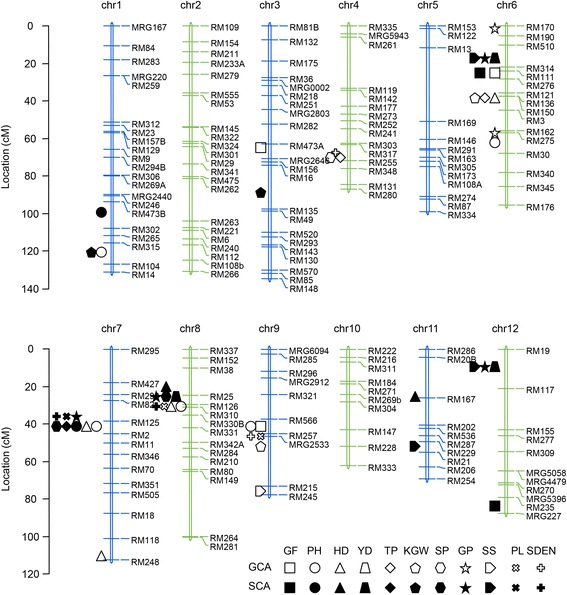
The QTLs detected for GCA and SCA effect in NCII population. For a description of agronomic traits see materials and methods

We applied linkage analysis to both phenotypic value and GCA effect of 190 DHs for 11 agronomic traits and identified many known and unknown QTLs for these traits (Fig. 3 and Additional file 1: Figure S1). Most loci detected in analysis of phenotype were also detected in GCA and we found these QTLs often explained larger proportion of phenotypic variations in DH population. Several QTLs for PH and HD also contribute to YD in DHs, such as *Hd1* and *Ghd8* (Yan et al. 2011; Yano et al. 2000). However, they have no effect on GCA and we detected no QTL for YD in analysis of GCA. This may be due to the low *h* ^*2*^_*N*_ of YD in our NCII population. Locations of QTLs for GCA on linkage map are show in Fig. 4 and detailed information is show in Additional file 2: Table S1.

We further performed linkage analysis to both phenotype and SCA effect for two BC populations (ZS97 × DHs and WYG × DHs) and indentified 66 QTLs for 11 agronomic traits. Most QTLs identified in BC populations were identified in DHs but with lower significance. Two new QTLs for SS and YD were detected on chromosome 6 and chromosome 12 in both phenotypic value and SCA effect of both BC populations. The QTL on chromosome 6 cover the known hybrid sterility gene *S5* and has similar genetic effect (Yang et al. 2012). QTL spanning *Ghd8* were identified for SP and YD in both phenotypic value and SCA effect of both BC populations. By sequencing these genes in ZS97 and WYG (Additional file 1: Figure S1), we found that ZS97 carried a *S5-i* allele of *S5* and non-functional allele of *Ghd8*, and WYG carried a *S5-j* allele of *S5* and functional allele of *Ghd8* (Yan et al. 2011; Yang et al. 2012). So the two QTLs for SCA of grain yield are actually *S5* and *Ghd8*. And the QTL on chromosome 12 is a new QTL for SS and YD. The location and detailed information for these QTLs are show in Fig. 4 and Additional files 3 and 4: Tables S2 and S3.

### Three QTLs for SCA effect of grain yield

**Fig. 5:**
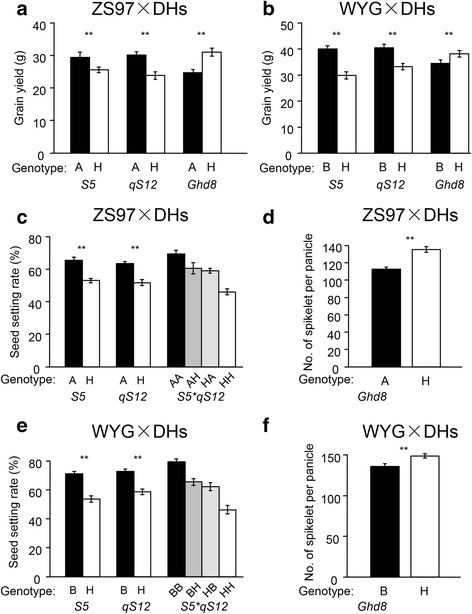
The effects of *S5*, *qS12* and *Ghd8* on agronomic traits in two BC populations. **a** The average grain yield of ‘A’ and ‘H’ genotype of the three QTLs in ZS97 × DHs; **b** The average grain yield of ‘B’ and ‘H’ genotype of the three QTLs in WYG × DHs; **c** The average seed setting rate of ‘A’ and ‘H’ genotype and combination genotype of *S5* and *qS12* in ZS97 × DHs; **d** The average spikelet number per panicle of ‘A’ and ‘H’ genotype of *Ghd8* in ZS97 × DHs; **e** The average seed setting rate of ‘B’ and ‘H’ genotype and combination genotype of *S5* and *qS12* in WYG × DHs; **f** The average spikelet number per panicle of ‘B’ and ‘H’ genotype of *Ghd8* in WYG × DHs; ‘A’ and ‘B’ represent the different homozygous allelic type from ZS97 and WYG, respectively. ‘H’ represents the hybrid allelic type of the two parents

We analyzed the effects of *S5*, *qS12* and *Ghd8* on grain yield in both BC populations and identified the overdominant effect of these QTLs. In ZS97 × DHs (Fig. 5a), heterozygous genotypes (H) of *S5* and *qS12* both show lower grain yield than correspondent homozygous ZS97 genotype (A); similarly in WYG × DHs (Fig. 5b), heterozygous genotypes (H) of *S5* and *qS12* both show lower grain yield than correspondent homozygous WYG genotype (B). This indicates *S5* and *qS12* show hybrid weakness in grain yield. On the contrary, the heterozygous genotype of *Ghd8* show higher grain yield than both homozygous genotypes and that indicates *Ghd8* show hybrid vigour in grain yield (Fig. 5a, b).

### Dosage effects of QTLs on yield traits.

**Fig. 6:**
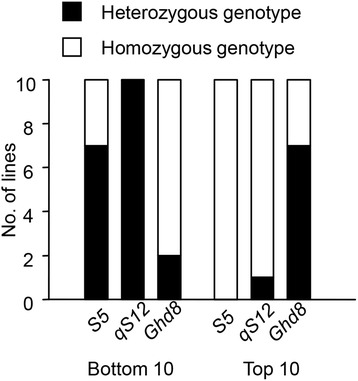
The advantage genotypes of *S5*, *qS12* and *Ghd8* were accumulated in the top 10 lines and the disadvantage genotypes were accumulated in the bottom 10 lines. For *S5* and *qS12*, homozygous genotypes are advantageous. For *Ghd8*, heterozygous genotype is advantageous

*S5* and *qS12* both effected on SS and YD in our NCII population and they also show dosage effect on SS and YD (Fig. 5c, e). The both homozygous genotype (AA or BB) show much higher SS and YD than the both heterozygous genotype (HH). The heterozygous genotype of *Ghd8* has more spikelet number than homozygous genotype which lead to the heterozygous advantage in grain yield (Fig. 5d, f). Though the genetic effect of *Ghd8* is different to *S5* and *S12*, the three genes show dosage effect on YD. Comparing the top 10 and bottom 10 lines of grain yield in BC populations, we found that advantage genotypes were accumulated in the top 10 lines and disadvantage genotypes were accumulated in the bottom 10 lines (Fig. 6). The F_1_ of ZS97 × WYG has heterozygous genotypes in all *S5*, *S12* and *Ghd8*, and the weakness in seed setting rate weight more than the heterosis in total spikelet number and grain weight (Table 1).

## Discussion

In this study, GCA and SCA effects in an *indica-japonica* cross were estimated using a NCII population. It is interesting to note that, the performance of GCA was not significantly or strongly (−0.35 < *r* < 0.35) correlated to the performance of DHs per se in several traits (Table 2). And these traits all have low heritability in our NCII population (Table 3). So traits with low heritability, as GF, YD and SS, are hardly to be predicted by parental performance. Weak correlations between the performance of inbred lines and their GCA effects were also detected in a previous study (Lv et al. 2012), so the performance of testcrosses was important for the evaluation and selection of elite inbred lines. On the other hand, only QTLs with large effects on agronomic traits can pass their effects from parents to hybrids. The major QTLs, *sd1*, *Ghd8* and *DEP1* (Sasaki et al. 2002; Yan et al. 2011; Yano et al. 2000), detected in DHs were also major QTLs for GCA effects (Fig. 3). However, several QTLs (*Hd1*, *Ghd7* and *Hd2*) have large effects in DHs but have little effects in GCA effects (Koo et al. 2013; Xue et al. 2008; Yano et al. 2000). And other QTLs have little effects in DHs can hardly be detected in GCA effects. These differences among QTLs also reflect the different genetic basis between parental performance and GCA.

**Table 2:** Phenotypic correlation (*r*) coefficients for agronomic traits in DHs and TC populations

*Correlations*	GF	PH	HD	YD	TP	KGW	SP	GP	SS	PL	SDEN
DHs and GCA_DHs_	0.32^**^	0.86^**^	0.75^**^	0.06	0.36^**^	0.55^**^	0.66^**^	0.38^**^	0.12	0.67^**^	0.73^**^
ZS97 × DHs and DHs	0.34^**^	0.83^**^	0.83^**^	0.42^**^	0.40^**^	0.58^**^	0.69^**^	0.57^**^	0.36^**^	0.69^**^	0.79^**^
ZS97 × DHs and GCA_DHs_	0.78^**^	0.89^**^	0.83^**^	0.50^**^	0.82^**^	0.77^**^	0.68^**^	0.47^**^	0.48^**^	0.87^**^	0.74^**^
ZS97 × DHs and SCA_ZS97×DHs_	0.78^**^	0.52^**^	0.44^**^	0.80^**^	0.73^**^	0.40^**^	0.57^**^	0.70^**^	0.79^**^	0.38^**^	0.42^**^
WYG × DHs and DHs	0.23^*^	0.60^**^	0.44^**^	−0.31^**^	0.22^*^	0.44^**^	0.31^**^	−0.17^*^	−0.16^*^	0.56^**^	0.34^**^
WYG × DHs and GCA_DHs_	0.52^**^	0.76^**^	0.48^**^	0.67^**^	0.64^**^	0.65^**^	0.68^**^	0.58^**^	0.67^**^	0.71^**^	0.69^**^
WYG × DHs and SCA_WYG×DHs_	0.74^**^	0.17^*^	0.17^*^	0.81^**^	0.62^**^	0.33^**^	0.50^**^	0.74^**^	0.85^**^	0.25^**^	0.48^**^
P64S × DHs and DHs	0.25^**^	0.82^**^	0.57^**^	−0.05	0.22^*^	0.58^**^	0.43^**^	0.33^**^	0.11	0.62^**^	0.59^**^
P64S × DHs and GCA_DHs_	0.73^**^	0.91^**^	0.88^**^	0.62^**^	0.70^**^	0.73^**^	0.79^**^	0.64^**^	0.59^**^	0.87^**^	0.83^**^
P64S × DHs and SCA_P64S×DHs_	0.63^**^	0.33^**^	0.61^**^	0.58^**^	0.66^**^	0.22^*^	0.65^**^	0.46^**^	0.24^*^	0.31^**^	0.63^**^
GCA_DHs_ and SCA_ZS97×DHs_	0.22^*^	0.21^*^	0.17^*^	−0.08	0.24^*^	0.02	−0.08	−0.10	−0.11	0.03	−0.13
GCA_DHs_ and SCA_WYG×DHs_	−0.20^*^	−0.35^**^	−0.56^**^	0.19^*^	−0.18^*^	−0.15^*^	−0.15^*^	0.08	0.29^**^	−0.26^**^	−0.15^*^
GCA_DHs_ and SCA_P64S×DHs_	−0.06	0.10	0.36^**^	−0.21^*^	−0.07	−0.11	0.24^*^	−0.07	−0.45^**^	0.00	0.26^**^

GCA_DHs_ denote the general combining ability of DH lines; SCA_ZS97×DHs_, SCA_WYG×DHs_ and SCA_P64S×DHs_ indicate the special combining ability for hybrids of ZS97 × DHs, WYG × DHs and P64S × DHs

*GF* grian-filling degree, *PH* plant height, *HD* heading date, *YD* yield, *TP* number of tillers per panicle, *KGW* grain weight per 1000 grains, *SP* spikelet per panicle, *GP* number of grains per panicle, *SS* seed-setting rate, *PL* panicle langth, *SDEN* grain density per panicle

^*^ *P* < 0.05, ^**^ *P* < 0.01

**Table 3:** Variance analysis of combining ability and genetic parameters estimation of agronomic traits

Trait	DHs	Testers	Testers × DHs	V_A_	V_Na_	*h* ^*2*^_*N*_
GF	37^**^	193^**^	27^**^	1.4	9.15	0.13
PH	1116^**^	18515^**^	230^**^	138.01	76.67	0.64
HD	924^**^	47864^**^	366^***^	164.84	121.91	0.57
YD	326^**^	8498^**^	392^**^	10.19	130.64	0.07
TP	35^**^	157^**^	19^**^	2.03	6.33	0.24
KGW	25^**^	3626^**^	9^**^	9.57	3.13	0.75
SP	3866^**^	140348^**^	2155^**^	489.26	718.35	0.41
GP	2308^**^	183467^**^	2793^**^	337.13	931.13	0.27
SS	618^**^	21437^**^	846^**^	19.26	281.87	0.06
PL	23^**^	823^**^	6^**^	3.68	1.89	0.66
SDEN	11^**^	419^**^	5^**^	1.57	1.59	0.5

*V*_*A*_, additive variance, *V*_*Na*_ non-additive variance, h^2^_N_ narrow-sense heritability, *GF* grian-filling degree, *PH* plant height, *HD* heading date, *YD* yield, *TP* number of tillers per panicle, *KGW* grain weight per 1000 grains, *SP* spikelet per panicle, *GP* number of grains per panicle, *SS* seed-setting rate, *PL* panicle langth, *SDEN* grain density per panicle

^**^ *P* < 0.01

*Indica-japonica* cross were reported to has higher heterosis than *indica-indica* cross and *japonica-japonica* cross (Yuan 1994). Although *indica* generally has better yield performance, *japonica* carries many beneficial alleles that are uncommon in *indica* gene pools (for example, *DEP1*, *IPA1* and *NAL1*) (Fujita et al. 2013; Huang et al. 2009; Jiao et al. 2010). Positive partial dominance and overdominance effects have served as the major causes of heterosis in *indica-japonica* F_1_ hybrids (Huang et al. 2016). Matting design in classic genetics helps to dissect the contributions of different genetic effects and QTL mapping in molecular genetics helps to identify important loci for hybrid performance (George 2012; Zeng 1994). We combined both methods of classic and molecular genetics to detect alleles of additive and non-additive effects contribute to inter-subspecies hybrid performance. This new method is better than simply perform QTL mapping to hybrid performance, as we identified more loci with higher significances and more clear effects (Additional files 2, 3, and 4: Tables S1-S3). For example, *sd1* is the major locus with additive effects (a = −7.54) on plant height of hybrids and *Ghd8* is the major locus with overdominace effect on grain yield of hybrids (Sasaki et al. 2002; Yan et al. 2011). The same locus may have different effect on different traits as *Ghd8* is also a major locus with additive effect (a = −9.42) on heading date of hybrids. The *S5* (Yang et al. 2012) and *qS12* are two loci conferring the wide-compatibility between *indica* and *japonica*. The two QTLs will accelerate the development of intersubspecific hybrids with high heterosis.

Prediction of hybrid performance is important in hybrid rice breeding. Heterosis and combining ability are two main indexes for hybrid performance. In this study, we dissect the hybrid performance of ZS97 × WYG and the NCII population in to GCA and SCA effects (Table 3). Among 11 agronomic traits, PH, HD, KGW and PL were mainly controlled by GCA effects, and major QTLs were detected for these traits (*sd1*, *Ghd8*, *qKGW2* and *DEP1* (Che et al. 2015; Huang et al. 2009; Sasaki et al. 2002; Yan et al. 2011)) (Figs. 3 and 4). On the contrary, GF, YD and SS were mainly controlled by SCA effects, and three QTLs (*S5*, *qS12* and *Ghd8*) contributed to these traits (Fig. 4). With the knowledge of these information, we will be able to predict the hybrid performance of an *indica-japonica* cross.

## Conclusion

We dissected the effects of GCA and SCA in an *indica-japonica* cross and identified lots of known and unknown QTLs for them. Among these QTLs, *Ghd8*, *S5* and *qS12* largely contributed to the hybrid grain yield of *indica-japonica* cross. These results provide insights into the genetic bases of combining ability and heterosis and will provide valuable information for the improvements of *indica-japonica* hybrid breeding.

## Methods

### Materials and field planting

The NCII and NCIII populations in our study were constructed according to the North carolina design II and III (George 2012). In a NCII design, each member of a group of parents used as males is mated to each member of another group of parents used as females. NCII design is used to evaluate GCA for inbred lines and SCA for every cross. In a NCIII design, a random sample of F_2_ plants (as well as RILs and DH lines) is backcrossed to the two inbred lines from which the F_2_ was descended.

Our study included 3 rice mapping populations (Fig. 1). The first one is a set of 190 Doubled haploid lines (DHs) derived from the anther culture of the F_1_ from a cross between an *indica* variety ZS97 and a *japonica* variety WYG (Jiang et al. 2004). Subsequently, taking the two parents as two testers, two BC populations were developed by crossing all 190 DHs to each parent. The gather of the two BC populations is also called a NCIII population. We took an *indcia* variety P64S as the third tester, and cross all 190 DHs to P64S. Finally, the NCII population was composed of the three testcross (TC) populations. The parents of the DHs (ZS97 and WYG), the F_1_ hybrid (ZS97 × WYG), were used as control.

The phenotypic performance was evaluated at the experimental field of Huazhong Agricultural University, Wuhan, China. All materials for these populations and parents were sown in the seedling nursery, and 27-day-old seedlings were transplanted into tow-row plots, 10 plants with 16.5 cm of space between plants with a row and 26.4 cm of space between the rows. The plots were arranged in a randomized complete block design with two replications.

### Trait measurements and statistical analyses

Agronomic traits measured in this study included grain yield per plant (YD), number of tillers per plant (TP), number of grains per panicle (GP), 1000-grain weight (KGW), spikelet per panicle (SP), panicle length (PL), grain density per panicle (SDEN), grain-filling degree (GF), and seed-setting rate (SS). Trial means were determined from eight randomly selected plants in the middle of the rows of all lines. The former four traits, YD, TP, GP, and KGW, were essentially as described previously by Yu et al. (1997). The SP was scored as the total number of spikelets divided by the number of reproductive tillers of a plant. The PL was measured as the average length from the bottom neck of three main panicles to their tips for each plant. The SDEN was scored as the number of grains divided by the PL, with average grain number per centimeter for three main panicles representative of each plant. The GF was scored as the percentage of the average weight of a single fertilized grain compared to the weight of single grain with mass density > 1 in each plant, essentially as previously described by Niu et al. (2004) and Zhu et al. (1995). SS was scored as the number of grains divided by the total number of spikelets from the reproductive tillers of a plant. Phenotypic distribution of 11 agronomic traits were drew by boxplot() function of R software (Ihaka and Gentleman 1996). *P*-values for phenotypic coefficients were calculated with a two-sided *t-test* using cor.test() function in R.

### Genotyping and linkage map construction

A linkage map consisting of 179 SSR markers (Fig. 4) covering the 12 chromosomes with a total length of 1849.4 cM (an average length of 9.4 cM) between two markers was constructed as described by Jiang et al. (2004). All the 179 markers are public makers and their sequence can be queried on GRAMENE website (http://www.gramene.org/).

### Variance analysis and QTL mapping

Two-factor variance analysis were performed to the NCII population to calculated the effects of DHs, testers and DHs × testers. The GCA variance effects of the parents and the SCA variance effects of the hybrids were estimated by the fixed model described by Mo (1982).

The mathematical representation of the relationship between phenotype and combining ability for each cross is: Y_*ij*_ =‾Y + G_*i*_ + G_j_ + S_*ij*_; where Y_*ij*_ is the phenotype value of the hybrid derived from the *i* th male parent and *j* th female parent,‾Y is the mean phenotype value of all hybrids, G_*i*_ is the general combining ability (GCA) of the *i* th male parent, G_*j*_ is the GCA of the *j* th female parent, S_*ij*_ is the specific combining ability (SCA) of the hybrid derived from the *i* th male parent and the *j* th female parent. As Y_*i*_ is the mean phenotype of the hybrid derived from the *i* th male parent and Y_*j*_ is the mean phenotype of the hybrid derived from the *j* th female parent, the combining ability was calculated by the following equations:

$$ {\mathrm{G}}_i={\overline{\mathrm{Y}}}_i-\overline{\mathrm{Y}};{\mathrm{G}}_j={\overline{\mathrm{Y}}}_j-\overline{\mathrm{Y}};{\mathrm{S}}_{ij}={\overline{\mathrm{Y}}}_{ij}-{\overline{\mathrm{Y}}}_i-{\overline{\mathrm{Y}}}_j-\overline{\mathrm{Y}} $$

QTL analysis was performed separately for the DH and BC populations. For the DH population, the mean trait values and GCA effect were used as input data. For each of the BC populations, the mean trait values and SCA effect of the BC hybrids were used as input data. The SCA data corresponding to the ZSF1 population were referred to as SCA_ZS97_, and the data from the WYF1 population were referred to as SCA_WYG_. Analyses of main-effect QTL (M-QTL) was conducted in each mapping population by composite interval mapping using WinQTLCart version 2.5 software (Zeng 1994). In the analyses, the likelihood ratio (LR) and *t* test were combined to test the significance of the single-locus QTL additive effects. The LR and t values corresponding to *P* = 0.001, equivalent to LOD (log likelihood value) ≥3.0 threshold according to calculated results, were used as the threshold for claiming the putative M-QTLs. The peak points of the LR in the linkage map were considered the putative positions of the QTLs. The relative contribution of a genetic component (*R* ^*2*^; %) was calculated as the proportion of phenotypic variance explained by that component in the selected model.

### Candidate gene confirmation

Many QTLs were colocated with known genes and have similar effects on agronomic traits. To confirm whether these QTLs were identical to these cloned genes, we sequenced these genes in ZS97B and Wuyugeng2 and compared their allelotype (Additional file 1: Figure S1) according to previous studies (Huang et al. 2009; Kojima et al. 2002; Koo et al. 2013; Sasaki et al. 2002; Xue et al. 2008; Yan et al. 2011; Yang et al. 2012; Yano et al. 2000).

## Additional files

Additional file 1: Figure S1. Allele differences between Wuyugeng2 and Zhenshan97B in 8 cloned genes. Genes structure of *sd1* (**a**), *Hd3a* (**b**), *S5* (**c**), *Hd1* (**d**), *Ghd7* (**e**), *Hd2* (**f**), *Ghd8* (**g**) and *dep1* (**h**) in Wuyugeng2 and Zhenshan97B. (TIFF 305 kb)

Additional file 2: Table S1. Detailed information for QTLs detected in DHs and GCA effects. (DOCX 18 kb)

Additional file 3; Table S2. Detailed information for QTLs detected in ZS97 × DHs and SCA effects. (DOCX 16 kb)

Additional file 4: Table S3. Detailed information for QTLs detected in WYG × DHs and SCA effects. (DOCX 16 kb)

## Abbreviations

DHs: double haploid lines
GCA: general combining ability
QTLs: quantitative trait locus
SCA: specific combining ability
