# Supplementary figures and images for: Dissecting combining ability effect in a rice NCII-III population provides insights into heterosis in indica-japonica cross
Source: Rice (N Y). 2017 Aug 29;10:39. doi: 10.1186/s12284-017-0179-9 (PMC5574824; doi:10.1186/s12284-017-0179-9)

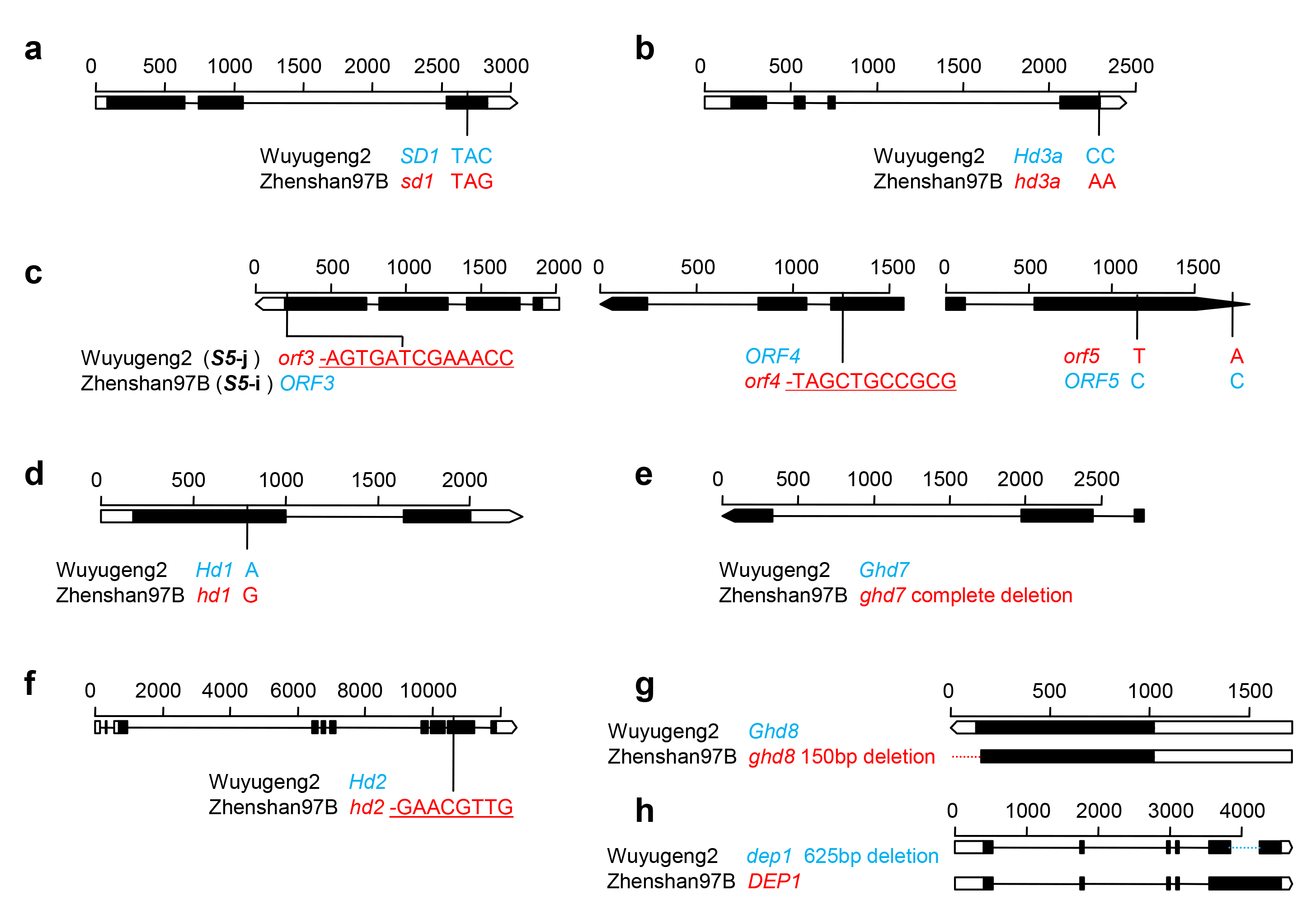

Supplement: Supplementary file 1 — Allele differences between Wuyugeng2 and Zhenshan97B in 8 cloned genes. Genes structure of sd1 (a), Hd3a (b), S5 (c), Hd1 (d), Ghd7 (e), Hd2 (f), Ghd8 (g) and dep1 (h) in Wuyugeng2 and Zhenshan97B. (TIFF 305 kb) [file 12284_2017_179_MOESM1_ESM.tif]
