# Supplementary material for: Dissecting combining ability effect in a rice NCII-III population provides insights into heterosis in indica-japonica cross
Source: Rice (N Y). 2017 Aug 29;10:39. doi: 10.1186/s12284-017-0179-9 (PMC5574824; doi:10.1186/s12284-017-0179-9)
Supplement: Supplementary file 2 — Detailed information for QTLs detected in DHs and GCA effects. (DOCX 18 kb) [file 12284_2017_179_MOESM2_ESM.docx]

**Additional file 1: Table S1.** **Detailed information for QTLs detected in DHs and GCA effects.**

| Trait | Chr | Interval | DHs | | |  | GCA_DHS_ | | |
| --- | --- | --- | --- | --- | --- | --- | --- | --- | --- |
|  |  |  | LOD | A | *R^2^* |  | LOD | A | *R^2^* |
| GF | 3 | MRG2646-RM135 | 5.57 | 1.71 | 0.14 |  | 3.46 | 0.96 | 0.08 |
| GF | 6 | RM314-RM121 |  |  |  |  | 3.34 | -0.69 | 0.07 |
| GF | 9 | RM566-RM257 |  |  |  |  | 3.73 | 0.71 | 0.08 |
| PH | 1 | RM302-RM104 | 15.87 | -10.74 | 0.30 |  | 19.07 | -7.54 | 0.32 |
| PH | 3 | MRG2646-RM135 | 3.39 | 4.10 | 0.04 |  | 3.02 | 2.63 | 0.04 |
| PH | 6 | RM276-RM136 | 3.33 | 4.30 | 0.05 |  | 4.06 | 3.29 | 0.06 |
| PH | 7 | RM125-RM346 | 6.43 | -6.15 | 0.10 |  | 4.70 | -3.40 | 0.07 |
| PH | 8 | RM25-RM331 | 6.68 | -6.20 | 0.10 |  | 8.23 | -4.93 | 0.13 |
| PH | 9 | RM566-RM257 | 6.06 | 6.35 | 0.11 |  | 8.75 | 4.95 | 0.13 |
| HD | 3 | RM135-RM293 | 4.53 | 3.77 | 0.06 |  |  |  |  |
| HD | 6 | RM170-RM190 | 4.44 | 3.62 | 0.05 |  |  |  |  |
| HD | 6 | RM121-RM162 | 6.56 | 4.55 | 0.08 |  | 6.21 | 3.48 | 0.08 |
| HD | 7 | RM125-RM346 | 18.14 | -9.40 | 0.35 |  | 10.60 | -4.90 | 0.15 |
| HD | 7 | RM118-RM248 | 12.50 |  |  |  |  |  |  |
| HD | 8 | RM25-RM331 | 13.43 | -7.54 | 0.23 |  | 31.01 | -9.42 | 0.55 |
| YD | 6 | RM121-RM162 | 3.79 | 2.19 | 0.07 |  |  |  |  |
| YD | 8 | RM25-RM331 | 5.71 | -2.78 | 0.12 |  |  |  |  |
| YD | 11 | RM21-RM254 | 4.19 | 2.43 | 0.09 |  |  |  |  |
| TP | 4 | RM303-RM348 | 4.08 | 0.97 | 0.08 |  | 3.01 | 0.55 | 0.06 |
| TP | 6 | RM121-RM162 | 3.78 | -0.95 | 0.07 |  | 3.87 | -0.63 | 0.08 |
| KGW | 2 | RM145-RM341 | 5.74 | -1.07 | 0.10 |  |  |  |  |
| KGW | 3 | MRG2646-RM135 | 3.27 | 0.68 | 0.05 |  |  |  |  |
| KGW | 6 | RM121-RM162 |  |  |  |  | 4.07 | -0.60 | 0.08 |
| KGW | 9 | RM566-RM257 | 9.25 | 1.21 | 0.18 |  | 4.48 | 0.73 | 0.12 |
| KGW | 12 | RM235-MRG227 | 3.68 | -0.80 | 0.07 |  |  |  |  |
| SP | 4 | RM303-RM348 |  |  |  |  | 5.13 | -7.73 | 0.10 |
| SP | 6 | RM121-RM162 | 9.61 | 12.36 | 0.17 |  | 6.92 | 9.48 | 0.15 |
| SP | 7 | RM125-RM346 | 5.41 | -9.33 | 0.10 |  |  |  |  |
| SP | 8 | RM38-RM25 | 7.60 | -12.30 |  |  |  |  |  |
| GP | 6 | RM170-RM190 |  |  |  |  | 3.43 | -5.32 | 0.07 |
| GP | 6 | RM121-RM162 | 6.38 | 9.73 | 0.12 |  | 4.97 | 7.00 | 0.12 |
| GP | 8 | RM38-RM25 | 6.80 | -10.18 | 0.13 |  |  |  |  |
| SS | 9 | RM215-RM245 |  |  |  |  | 4.29 | -3.27 | 0.10 |
| PL | 6 | RM121-RM162 | 4.76 | 0.84 | 0.09 |  |  |  |  |
| PL | 7 | RM351-RM18 | 4.91 | -0.85 | 0.09 |  |  |  |  |
| PL | 8 | RM38-RM25 | 4.76 | -0.84 | 0.09 |  | 4.54 | -0.51 | 0.07 |
| PL | 9 | RM566-RM257 | 17.93 | 1.71 | 0.36 |  | 18.76 | 1.15 | 0.35 |
| PL | 12 | RM19-RM117 | 4.33 | 0.91 | 0.10 |  |  |  |  |
| SDEN | 4 | RM303-RM348 |  |  |  |  | 3.38 | -0.31 | 0.05 |
| SDEN | 6 | RM121-RM162 | 5.98 | 0.50 | 0.09 |  | 4.81 | 0.40 | 0.09 |
| SDEN | 7 | RM125-RM346 | 4.70 | -0.45 | 0.07 |  |  |  |  |
| SDEN | 8 | RM25-RM331 | 6.16 | -0.53 | 0.09 |  |  |  |  |
| SDEN | 9 | RM566-RM257 | 14.87 | -0.87 | 0.25 |  | 9.69 | -0.59 | 0.20 |

Chr, chromosome; DHs, double haploid lines; GCA_DHs_, GCA effects in DHs; LOD, log likelihood value; A, additive effect.
