# Supplementary material for: Dissecting combining ability effect in a rice NCII-III population provides insights into heterosis in indica-japonica cross
Source: Rice (N Y). 2017 Aug 29;10:39. doi: 10.1186/s12284-017-0179-9 (PMC5574824; doi:10.1186/s12284-017-0179-9)
Supplement: Supplementary file 3 — Detailed information for QTLs detected in ZS97 × DHs and SCA effects. (DOCX 16 kb) [file 12284_2017_179_MOESM3_ESM.docx]

**Additional file 2: Table S2****. Detailed information for QTLs detected in ZS97×DHs and SCA effects.**

| Trait | Chr | Interval | ZS97×DHs | | |  | SCA_WYG×DHs_ | | |
| --- | --- | --- | --- | --- | --- | --- | --- | --- | --- |
|  |  |  | LOD | A | R2 |  | LOD | A | R2 |
| GF | 6 | RM314-RM121 | 6.70 | -3.07 | 0.16 |  | 4.42 | -1.79 | 0.10 |
| GF | 12 | RM235-MRG227 | |  |  |  | 3.65 | -1.66 | 0.09 |
| PH | 1 | RM302-RM104 | 19.48 | -18.69 | 0.33 |  |  |  |  |
| PH | 7 | RM125-RM346 | 12.72 | -13.51 | 0.18 |  | 4.57 | -6.27 | 0.13 |
| PH | 8 | RM25-RM331 | 9.44 | -12.19 | 0.14 |  |  |  |  |
| PH | 9 | RM566-RM257 | 9.60 | 12.28 | 0.14 |  |  |  |  |
| HD | 7 | RM125-RM346 | 20.65 | -19.61 | 0.42 |  |  |  |  |
| HD | 8 | RM25-RM331 | 15.48 | -15.65 | 0.28 |  |  |  |  |
| YD | 6 | RM170-RM314 | |  |  |  | 3.97 | 7.50 | 0.12 |
| YD | 7 | RM125-RM346 | 5.75 | -7.98 | 0.13 |  | 3.02 | -5.83 | 0.07 |
| YD | 8 | RM25-RM331 | 4.54 | -6.99 | 0.10 |  | 4.53 | -6.98 | 0.11 |
| YD | 12 | RM19-RM117 | 4.05 | 7.38 | 0.11 |  | 6.20 | 8.98 | 0.19 |
| TP | 7 | RM125-RM346 | |  |  |  | 3.10 | 1.15 | 0.08 |
| KGW | 6 | RM314-RM121 | 6.80 | -1.89 | 0.14 |  |  |  |  |
| KGW | 9 | RM566-RM257 | 4.78 | 1.68 | 0.11 |  |  |  |  |
| KGW | 12 | RM235-MRG227 | 3.92 | -1.50 | 0.09 |  |  |  |  |
| SP | 4 | RM303-RM348 | 9.40 | -25.06 | 0.17 |  |  |  |  |
| SP | 7 | RM125-RM346 | 9.90 | -26.07 | 0.19 |  | 6.96 | -21.32 | 0.16 |
| SP | 8 | RM25-RM331 | 9.77 | -34.03 | 0.17 |  | 6.59 | -21.74 | 0.16 |
| GP | 6 | RM314-RM121 | 5.31 | 19.41 | 0.10 |  | 4.61 | 24.12 | 0.14 |
| GP | 7 | RM125-RM346 | 8.63 | -25.05 | 0.18 |  | 3.28 | -17.95 | 0.08 |
| GP | 8 | RM25-RM331 | 5.12 | -18.88 | 0.10 |  | 3.83 | -19.33 | 0.09 |
| GP | 12 | RM19-RM117 | 3.35 | 16.64 | 0.08 |  | 3.68 | 20.48 | 0.11 |
| SS | 6 | RM170-RM314 | 6.54 | 13.65 | 0.16 |  | 8.11 | 15.76 | 0.21 |
| SS | 11 | RM202-RM21 |  |  |  |  | 5.02 | 11.17 | 0.10 |
| SS | 12 | RM19-RM117 | 4.35 | 11.19 | 0.11 |  | 6.08 | 12.54 | 0.15 |
| PL | 8 | RM25-RM331 | 6.46 | -1.55 | 0.10 |  |  |  |  |
| PL | 9 | RM566-RM257 | 18.91 | 3.03 | 0.43 |  |  |  |  |
| SDEN | 4 | RM303-RM348 | 6.75 | -1.04 | 0.13 |  |  |  |  |
| SDEN | 7 | RM125-RM346 | 8.16 | -1.16 | 0.16 |  | 3.80 | -0.82 | 0.10 |
| SDEN | 8 | RM25-RM331 |  |  |  |  | 4.88 | -0.99 | 0.14 |
| SDEN | 9 | RM566-RM257 | 5.02 | -0.96 | 0.11 |  |  |  |  |
| SDEN | 11 | RM286-RM167 | 3.38 | 0.68 | 0.06 |  |  |  |  |

Chr, chromosome; WYG×DHs, the F_1_ population of WYG and double haploid lines; SCA_WYG×DHs_, GCA effects in WYG×DHs; LOD, log likelihood value; A, additive effect.
