# Supplementary material for: Dissecting combining ability effect in a rice NCII-III population provides insights into heterosis in indica-japonica cross
Source: Rice (N Y). 2017 Aug 29;10:39. doi: 10.1186/s12284-017-0179-9 (PMC5574824; doi:10.1186/s12284-017-0179-9)
Supplement: Supplementary file 4 — Detailed information for QTLs detected in WYG × DHs and SCA effects. (DOCX 16 kb) [file 12284_2017_179_MOESM4_ESM.docx]

**Additional file 3: Table S3. Detailed information for QTLs detected in WYG×DHs and SCA effects.**

| Trait | Chr | Interval | WYG×DHs | | |  | SCA_WYG×DHs_ | | |
| --- | --- | --- | --- | --- | --- | --- | --- | --- | --- |
|  |  |  | LOD | A | R2 |  | LOD | A | R2 |
| GF | 6 | RM121-RM162 | |  |  |  | 4.27 | -2.99 | 0.10 |
| GF | 9 | RM215-RM245 | 3.35 | -2.09 | 0.12 |  |  |  |  |
| PH | 1 | RM302-RM104 | 5.90 | 8.08 | 0.16 |  | 5.72 | -7.33 | 0.15 |
| PH | 3 | RM473A-RM135 | 4.12 | -5.83 | 0.08 |  |  |  |  |
| PH | 5 | RM291-RM274 | |  |  |  | 3.58 | -5.09 | 0.07 |
| PH | 6 | RM162-RM340 | 8.12 | -9.43 | 0.22 |  |  |  |  |
| PH | 8 | RM25-RM331 | |  |  |  | 6.09 | -7.11 | 0.13 |
| PH | 9 | RM566-RM257 | 3.82 | -6.89 | 0.12 |  |  |  |  |
| HD | 3 | RM135-RM293 | 5.49 | -5.05 | 0.11 |  |  |  |  |
| HD | 6 | RM121-RM162 | 7.60 | -7.70 | 0.27 |  |  |  |  |
| HD | 8 | RM25-RM331 | |  |  |  | 13.18 | -16.31 | 0.34 |
| YD | 6 | RM170-RM314 | 3.46 | 8.22 | 0.08 |  | 6.32 | 8.88 | 0.16 |
| YD | 8 | RM25-RM331 | 3.64 | -13.60 | 0.09 |  | 4.45 | -12.26 | 0.11 |
| YD | 12 | RM19-RM117 | 4.87 | 9.17 | 0.12 |  | 5.40 | 7.73 | 0.13 |
| KGW | 1 | RM302-RM104 | |  |  |  | 4.07 | -2.25 | 0.13 |
| KGW | 3 | RM473A-RM135 | |  |  |  | 3.51 | 1.99 | 0.10 |
| KGW | 12 | RMG5058-RM235 | |  |  |  | 3.05 | -1.76 | 0.07 |
| SP | 2 | RM145-RM341 | 3.34 | -17.19 | 0.08 |  |  |  |  |
| SP | 6 | RM121-RM162 | 6.88 | -25.56 | 0.19 |  |  |  |  |
| GP | 6 | RM170-RM314 | 4.66 | 24.30 | 0.11 |  | 4.87 | 24.07 | 0.12 |
| GP | 8 | RM25-RM331 | 3.40 | -19.55 | 0.08 |  |  |  |  |
| GP | 12 | RM19-RM117 | 5.64 | 27.09 | 0.14 |  | 3.49 | 19.69 | 0.08 |
| GP | 12 | RMG5058-RM235 | 3.60 | 21.98 | 0.09 |  |  |  |  |
| SS | 3 | RM132-RM36 | 3.01 | 9.82 | 0.05 |  |  |  |  |
| SS | 6 | RM314-RM121 | 10.68 | 19.86 | 0.23 |  | 9.76 | 16.08 | 0.20 |
| SS | 12 | RM19-RM117 | 5.57 | 13.39 | 0.11 |  | 5.97 | 12.01 | 0.12 |
| SS | 12 | RM235-MRG227 | 3.37 | 10.54 | 0.07 |  |  |  |  |
| PL | 6 | RM162-RM340 | 4.36 | -0.95 | 0.10 |  |  |  |  |
| PL | 9 | RM566-RM257 | 4.95 | -1.05 | 0.12 |  |  |  |  |
| SDEN | 2 | RM145-RM341 | 3.15 | -0.89 | 0.07 |  |  |  |  |
| SDEN | 6 | RM121-RM162 | 5.18 | -1.19 | 0.15 |  |  |  |  |
| SDEN | 9 | RM566-RM257 | 3.92 | 0.98 | 0.10 |  | 3.30 | -1.00 | 0.13 |
